# The Social and Sexual Networks of Black Transgender Women and Black Men Who Have Sex with Men: Results from a Representative Sample

**DOI:** 10.1089/trgh.2018.0039

**Published:** 2018-12-18

**Authors:** Jerel M. Ezell, Matthew J. Ferreira, Dustin T. Duncan, John A. Schneider

**Affiliations:** ^1^ Section of Infectious Diseases and Global Health, University of Chicago Medical Center, Chicago, Illinois.; ^2^ Department of Sociology, University of Chicago, Chicago, Illinois.; ^3^ Chicago Center for HIV Elimination, University of Chicago, Chicago, Illinois.; ^4^ NYU Spatial Epidemiology Lab, Department of Population Health, NYU School of Medicine, New York, New York.; ^5^ Department of Public Health Sciences, University of Chicago, Chicago, IL.

**Keywords:** gender identity, HIV, men who have sex with men, respondent-driven sampling, social networks, transgender women

## Abstract

**Background:** Little research has evaluated the social and sexual network-related health outcomes of young black transgender women (TGW) or compared these outcomes with those of black men who have sex with men (MSM). Social network analysis offers one potent means of understanding the dynamics driving the broad spectrum of adverse outcomes experienced by these subgroups.

**Methods:** We examined the social and sexual health network traits of 618 black individuals assigned male at birth who have sex with men, 47 (7.6%) of whom identified as TGW. Using respondent-driven sampling, data collection occurred over three waves between 2013 and 2016, in Chicago, Illinois. Univariate, logistic regression, and confidant and sexual network analyses were conducted to characterize dynamic network features.

**Results:** TGW's mean age was 22.1 (standard deviation ±2.6). TGW's sexual networks were significantly less stable (stability ratio of 0.175 vs. 0.278 among MSM, *p*=0.03) and had greater network turnover (turnover ratio of 0.825 vs. 0.735, *p*=0.04). TGW also had significantly more sex partners (7.6 vs. 4.0, *p*=0.0002) and exchange sex (odds ratio=2.97; 95% confidence interval: 1.66–5.32, *p*<0.001), lower rates of employment (39.6% vs. 71.1%, *p*<0.001), and more reported an income <$20,000 (93.5% vs. 80.8%, *p*=0.029). Within confidant networks, TGW had a borderline significantly higher network turnover ratio (0.703 vs. 0.625, *p*=0.06). Furthermore, both TGW and MSM had high, but similar, HIV rates (42.3% vs. 30.6%, respectively; *p*=0.17). There were no significant structural network differences vis-à-vis mean degree (*p*=0.46), betweenness centrality (*p*=0.40), closeness centrality (*p*=0.18), or average shortest path length (borderline statistically significant at *p*=0.06).

**Conclusion:** Using data from a representative sample of younger black individuals, we observed black TGW have less sexual network stability in contrast to black MSM but comparable structural network features. We further observed that both groups, and black TGW especially, possess considerable system-level, socioeconomic, and sexual health burdens.

## Introduction

Recent analysis suggests that the current population of transgender individuals in the United States, persons whose gender identity differs from the sex which they were assigned at birth, numbers roughly 1 million,^1^ with changing definitions and greater acceptance for nonheteronormativity portending substantial increases in transgender population size estimates in coming years.^2^ However, researchers have only recently begun to systematically characterize contextual dynamics of transgender individuals, namely in the domain of health and long-term health outcomes. In spite of the limited scholarship in this space, studies have increasingly shown that transgender individuals have substantially higher rates of chronic disease and mental illness, and are less likely to access health care resources, in comparison with individuals in the general population.^3–5^

Salient, but limited, details exist in the literature on the stark sexual health risks among transgender women (TGW), a highly vulnerable contingent of the transgender population. A systematic review on the global epidemiology of HIV infection suggests that the transfeminine population has up to a 40% prevalence of laboratory-confirmed HIV.^6^ Relatedly, data captured in the United States illustrate that TGW possess the greatest HIV burden of any primary subgroup in the country, with an estimated 34.2-fold increased odds in relation to the general population.^7^

An additional gap in much of the extant literature on the health of TGW lies in its porous intersectional orientation,^8^ evidenced by a continued lack of robust data problematizing the stratification of health outcomes among those in this population from historically disadvantaged or disenfranchised group.^9^ In a population-based analysis of the 2014 Behavioral Risk Factor Surveillance System, transgender individuals, in comparison with nontransgender individuals, were found to be more likely to be nonwhite and to live below the poverty line (26.0% vs. 15.5%), and they were less likely to attend college (35.6% vs. 56.6%).^10^

Intersectionality refers to the dynamics in which multiple culturally reproduced disadvantages in one's life create iterative impacts on their health and well-being.^11–13^ Despite slow, but steady, overall gains in physical, sexual, and emotional health within sexual and gender minority communities, broad health disparities continue to loom large.^14,15^ In the context of these visible gendered and racialized dynamics, young black TGW possess a triply marginalized status, the effects of their gender identity and sexual preferences, which may vary widely, compounded by the impacts of racial discrimination and ostracization.^16–18^ The vista of HIV risk, and barriers to diagnosis and treatment, is especially dire among black TGW.^19^ An assessment of data from the National HIV Surveillance System demonstrated that there were 1002 HIV diagnoses among black TGW between 2009 and 2014, representing 51% of all cases among TGW^20^ compared with Hispanics and whites who comprised 29% and 11%, respectively, of all cases.

Interdisciplinary research is needed to clarify these aforementioned dynamics among young black TGW by exploring health and sociocultural outcomes in this population, including social and sexual networks. The majority of existing intersectional inquiry addressing black TGW has focused almost exclusively on inter-racial group differences. Little research has assessed intra-racial group differences, particularly among black persons assigned male at birth with variable gender identities and expressions.^21,22^ More recently, in a cross-sectional analysis conducted in Atlanta, researchers determined that, in comparison with black men who have sex with men (MSM), black TGW had lower HIV testing knowledge and a higher likelihood of having engaged in transactional sex.^23^ Similar patterns in sexual health disparities between black MSM and black TGW have also been observed elsewhere.^24,25^

Social and community stigma against nonheteronormative behavior, lower levels of education, and limited access to health-promoting resources typify the comparable highly salient challenges, which both black MSM and TGW may face and share.^21,26,27^ Furthermore, contextually unique social spaces such as the house/ballroom or gay family communities are unique to sexual and gender minority persons of color,^28,29^ both of whom are included in these unique social spaces. Research into the health and circumstances of black TGW has been, in part, stymied by methodological limits resulting from difficulties reaching the population, use of convenience and nonsystematically sampling approaches,^30^ and limited within-race gender comparisons.

Social and sexual networks are vital features of wellness, forecasting myriad health outcomes, including HIV risk.^31,32^ Social network analysis enables rich evaluation of social structure, mobility, and interactions between and among individuals in defined spaces.^33^ There is growing scholarship on the social networks of young black MSM (YBMSM) illustrating how their networks are structured and composed, serving as conduits through which not only adverse health and social risks radiate outward to individual members^21,34,35^ but also social support.^36^ However, little is known about network differences between young black TGW and black MSM. In consideration of the sparse base of YBMSM within-group analyses, and in particular often misclassified subgroups of populations of young black TGW, a clearer understanding of networks—in terms of size, density, and other contextual dimensions—may aid in the generation of interventions and policies to address the intricate social and health care needs of young black TGW. The purpose of this study was to contrast the social and sexual networks of young black TGW and black MSM, using a longitudinal representative sample in Chicago.

## Materials and Methods

### Research project overview: uConnect

The *uConnect* project is a representative cohort study in Chicago, which aims to explore and advance knowledge around the genesis and manifestations of social and sexual networks among young black individuals assigned male at birth who have sex with men.^37,38^ Data collection occurred over three waves from 2013 to 2016 with 18 months of follow-up included. This research design enabled longitudinal social network analysis resulting in the determination of dynamic features of the resulting networks. More details on the study design and sample generation have been previously described.^39^ In brief, recruitment and sample generation was facilitated through respondent-driven sampling (RDS).^40–43^ RDS is a method of participant recruitment that uses participants' social network relationships to cultivate a sample approximating a probability sample.^44,45^ In this context, RDS was carried out through a small number of seeds (*n*=62 who were highly socially connected individuals from the target communities).

Eligibility criteria was as follows: (1) self-identified as African American or black; (2) were assigned male gender at birth; (3) between 16 and 29 years old; (4) reported oral or anal sex with a male within the past 2 years; (5) spent the majority of their time on the South side of Chicago; and (6) willing to provide informed consent at the study visit. The study protocol was approved by the Institutional Review Board of NORC at the University of Chicago.

### Name generators

The uConnect interview consisted of a list of a maximum of five confidants for each respondent for whom follow-up information was collected. These questions on confidants appeared at the beginning of the interview after a short set of introductory questions: “In this next section, we will discuss your close social network, that is, the people with whom you discuss things that are important to you. So I can ask some follow-up questions, please list the names of the people with whom you discuss things that are important to you.”

The sex partner network generator was administered midway through the interview, where information on a maximum of six recent sexual partners was collected. After generating a list of the five most recent sexual partners, the respondent was asked an additional question about the current primary sexual partner.^46^ Respondents were asked to compare their list of confidants with the list of sex partners they had just named; any matches were then recorded. The confidant name generator used in Wave 1 was also used in Wave 2. After the confidant list was generated, the respondent was asked if any of the confidants in Wave 2 were the same as the confidants whom the respondent listed in Wave 1. Any matches indicated by the respondent were recorded.

The sex partner name generator used in Wave 2 was similar to that used in Wave 1, with the difference that the sex partner name generated in Wave 2 asked respondents to list sexual partners since the last interview and not the past 6 months. Respondents were asked to compare sex partners listed at Wave 2 with those listed at Wave 1 in the same manner as described for confidants. Any matches indicated by the respondents were recorded. A similar name generator procedure for both confidants and sex partners was used at Wave 3 with the difference that the confirmation list provided was cumulative.

### Social network analysis: confidant and sexual network assessment

Traditional network metrics utilized to characterize the sample included degree, betweenness centrality, closeness centrality, and average shortest path length^47,48^: *Degree* references the number of links a network participant has to other network individuals; *betweenness centrality* assesses the amount of times the shortest path between two other network individuals includes the focal participant; *closeness centrality* measures a network participant's proximity to other individuals in the network; and *average shortest path length* references the general (shortest) distance between network nodes.

The names and subsequent information of respondents' confidants and sexual partners were used to explore the networks of TGW and MSM in the sample. Several proportion variables were created by calculating the proportion of an individual respondent's confidant and/or sexual network satisfied a certain criterion (e.g., proportion of HIV-positive network members) as in previous work.^49^

A turnover ratio was calculated for the confidant and sexual networks of every respondent with two or more study visits.^39,50,51^ The turnover ratio was calculated as the total number of new and lost ties (i.e., connections between members) divided by the cumulative network size from all study visits. Similarly, a stability ratio was calculated for the confidant and sexual networks of ever respondent with two or more study visits.^39,52,53^ The stability ratio was calculated as the number of retained ties divided by the original network size.

### Survey data collection

We also collected demographic data, including information on sexual orientation, relationship status, education, income (dichotomized into <$20,000 or >$20,000), employment status, and housing stability. In addition, we captured contextual information, such as HIV status, health care insurance status, number of confidants, number of sexual partners, and whether or not the individual had ever been criminal justice involved.

### HIV testing

Laboratory testing was done to determine HIV infection status (including acute infection). Replicating prior work we utilized fourth-generation HIV immunoassay (Abbott ARCHITECT HIV Ag/Ab Combo assay), HIV-1/-2 Ab differentiation (Bio-Rad Multispot HIV-1/-2 Rapid Test), and viral load testing (Abbott ReaLTime HIV-1 assay) administered to eluted dry blood spot samples.^54^

### Data analysis

Descriptive analysis was performed to produce summary statistics. Totals, means, and standard deviations (SDs) were used to characterize the primary variables for both TGW and MSM. Baseline differences between TGW and MSM on the variables of interest were analyzed using independent *t*-tests. Logistic regression was conducted to examine the relationship between the primary dependent variables and independent variable of interest. Findings were deemed to be statistically significant at *p*≤0.05 (two-sided). All data were analyzed using Stata 14.2.

## Findings

### Characteristics of black TGW

A final sample of 618 individuals at Wave 1 was included in the analysis. Study retention between Waves 1 and 2 was higher (86.4%) for MSM than TGW (66.7%) in the sample. Complete sample details at Wave 1 are provided in Table 1. In brief, of those enrolled at Wave 1, 47 (7.6%) identified as TGW. The mean age of TGW at Wave 1 was 22.1 *±* 2.6 years. The majority of TGW had a high school degree or less (59.0%) or some college/an associate's degree (38.5%). Roughly 43.8% of TGW identified as gay, 33.3% identified as straight, and 16.7% identified as bisexual; this pattern represented a statistically significant difference as compared with MSM, where 68.4% identified as gay, 1.1% identified as straight, and 27.9% identified as bisexual. TGW exhibited significantly more self-identified changes to their sexual identity between Waves 1 and 2, with 68.8% of TGW identifying with the same sexual orientation at both waves compared with 83.6% of MSM.

**Table 1.** Sociodemographic and Behavioral Characteristics Among Young Black Transgender Women and Black Men Who Have Sex with Men (uConnect Wave 1, Chicago, IL, 2013–2014) (*n*=618)

	Black TGW (*n*=47, 7.6%)	Black MSM (*n*=571, 92.4%)	*p*
Age	22.1 (2.6)	22.8 (3.2)	0.15
Has health insurance	27 (58.7)	304 (54.8)	0.607
Sexual orientation			<0.001^*^
Gay	21 (43.8)	388 (68.4)	
Straight	16 (33.3)	6 (1.1)	
Bisexual	8 (16.7)	158 (27.9)	
Other	3 (6.3)	15 (2.7)	
HIV positive (laboratory confirmed)	11 (30.6)	199 (42.3)	0.17
In a romantic relationship	16 (33.3)	224 (39.9)	0.374
Education			0.492
High school or less	23 (59.0)	177 (46.6)	
Some college/associate's degree	15 (38.5)	171 (45.0)	
Bachelor's degree	1 (2.6)	26 (6.8)	
Post-bachelor's degree	0 (0.0)	6 (1.6)	
Employed	19 (39.6)	404 (71.1)	<0.001^*^
Income <$20,000/year	43 (93.5)	446 (80.8)	0.029^*^
Stably housed	33 (68.8)	427 (75.4)	0.304
Ever in jail	25 (52.1)	258 (45.5)	0.38
Number of partners (previous 6 months)	7.6 (15.2)	4.0 (4.9)	0.0002^*^

^*^ Statistically significant at *p*≤0.05.

MSM, men who have sex with men; TGW, transgender women.

Continuing, as compared with MSM, significantly fewer TGW reported being employed (39.6% vs. 71.1%, *p*<0.001) and significantly more had an annual income of <$20,000 (93.5% vs. 80.8%, *p*=0.029). There were no statistically significant differences in laboratory-confirmed HIV status between MSM and TGW. In addition, after controlling for transgender identity, at each successive wave, there were statistically fewer individuals with insurance and statistically more individuals indicating being unstably housed (data not shown).

During Wave 1, TGW reported significantly more sex partners (*p*=0.0002), in the previous 6 months, at 7.6 partners (SD ±15.2) in contrast to 4.0 partners (SD ±4.9) among MSM. This pattern remained and was statistically significant in Wave 2 but not Wave 3 (data not shown).

### Social networks

**FIG. 1.**
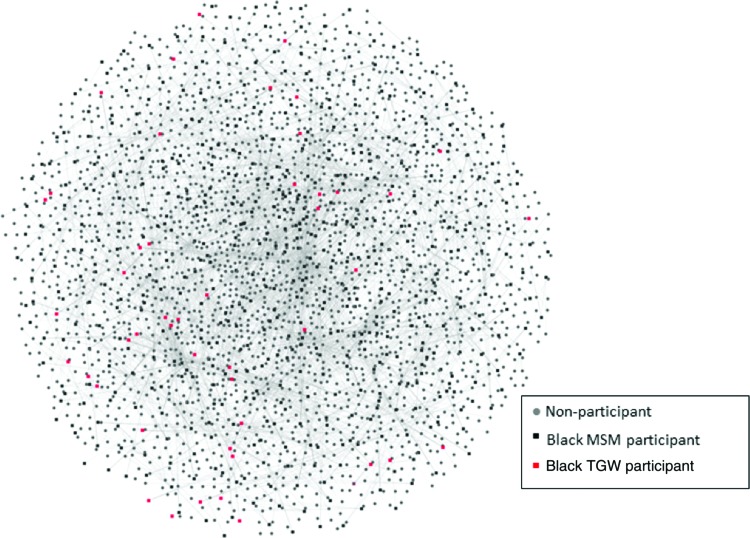
A visual representation of the young black MSM and TGW social network. (uConnect Study Wave 1, Chicago, Illinois, 2013–2014). MSM, men who have sex with men; TGW, transgender women.

A schematic of the entire social network is presented in Figure 1, with associated metrics outlined in Table 2. This network figure includes RDS, confidant, and sexual partner ties. There were no statistically significant differences in the applied structural network metrics—mean degree (*p*=0.46), betweenness centrality (*p*=0.40), closeness centrality (*p*=0.18), or average shortest path length that was borderline statistically significant (*p*=0.06)—between TGW and MSM in the cohort.

**Table 2.** Network Metrics of the Social Network for Young Black Transgender Women and Young Black Men Who Have Sex with Men (uConnect Study, Chicago, IL, 2013–2014)

	Black TGW	Black MSM	*p*
Degree	5.62	5.35	0.46
Betweenness centrality	0.63	0.58	0.40
Closeness centrality	0.66	0.59	0.18
Average shortest path length	2.29	3.09	0.06

As shown in Table 3, TGW had a significantly higher proportion of transgender confidants and a significantly higher number of transgender confidants who have sex with men when compared with MSM. Furthermore, TGW had a significantly lower proportion of male confidants (odds ratio [OR]=0.18, *p*<0.001) and significantly fewer male confidants who have sex with men (OR=0.56, *p*=0.002). In addition, TGW had fewer cis women sex partners (OR=0.29, *p*=0.017) and fewer sex partners whose sexual preference was men (OR=0.07, *p*<0.001) when compared with MSM. TGW had more sex partners with whom they exchanged sex for pay (OR=2.97, *p*<0.001); a higher number of sex partners from whom they received payment for sexual encounters (OR=3.6, *p*=0.02); a greater proportion of sex partners who exchanged sex for pay (OR=6.02, *p*<0.001); and a greater number of sex partners with a sexual preference for TGW (OR=23.9, *p*<0.001) and cis women (OR=2.91, *p*<0.001).

**Table 3.** Unadjusted Odds Ratios Relating Network Characteristics of Young Black Transgender Women and Black Men Who Have Sex with Men (uConnect Study Wave 1, Chicago, IL, 2013–2014)

	Unadjusted OR	*p*
Confidant networks		
Proportion of transgender confidants	58.4	<0.001^**^
Number of transgender confidants who have sex with men	7.92	<0.001^**^
Proportion of men confidants	0.18	0.002^**^
Number of men confidants who have sex with men	0.56	<0.001^*^
Number of HIV positive confidants	0.78	0.071
Sexual networks		
Exchange sex	2.97	<0.001^**^
Received payment for sex	3.6	0.02^*^
Partner prefers TGW	23.9	<0.001^**^
Partner prefers cis women	2.91	<0.001^**^
Proportion of partners who exchange sex for pay	6.02	<0.001^**^
Cis women partners	0.29	0.017^*^
Partner prefers men	0.07	0.07

Comparisons between black TGW and black MSM (reference group).

^*^ *p*<0.05.

^**^ *p*<0.001.

OR, odds ratio.

Within confidant networks (Table 4), TGW had a borderline significantly higher turnover ratio (0.703) as compared with MSM (0.625) (*p*=0.064). There were no statistically significant differences in the stability ratio. However, in sexual network analysis, TGW demonstrated a significantly lower stability ratio (0.175) than MSM (0.278) (*p*=0.03). In addition, TGW had a significantly higher turnover ratio (0.825) than MSM (0.735) (*p*=0.04).

**Table 4.** Sexual Partner and Confidant Network Stability of Young Black Transgender Women (uConnect Study Waves 1–3, Chicago, IL, 2013–2016)

	**Confidant network**		
	Black TGW	Black MSM	*p*
Stability ratio	0.334	0.408	0.103
Turnover ratio	0.703	0.625	0.064

	**Sexual network**		
	Black TGW	Black MSM	*p*
Stability ratio	0.175	0.278	0.0335^*^
Turnover ratio	0.825	0.735	0.0374^*^

^*^ Statistically significant at *p*≤0.05.

## Discussion

In this study, we applied an intersectional empirical approach to characterizing the social and sexual networks of black TGW, using a representative RDS-recruited sample of black individuals assigned male at birth who possess differing gender identities, have sex with men, and share similar sociocultural communities in Chicago's highly racially/ethnically segregated urban spaces. Our results illustrated that black TGW's sexual networks change significantly more than black MSM's and are generally less stable. These dynamics are especially critical given the observed association between belongingness, well-being, and sexual health in the transgender population.^55–57^ Moreover, we found that TGW have a significantly higher number of transgender confidants than MSM, yet have very similar structural network features in terms of degree and other sociostructural network metrics.

The pronounced morbidity that black TGW experience likely reflects, in part, the dense structural effects of high levels of economic deprivation, violence, and substandard schooling in majority-black communities *writ large*. Furthermore, black individuals often live, work, socialize, worship, and establish romantic and sexual partnerships in the same spaces^58^; little is known about whether these interactional tendencies are comparable or intensified among black TGW. However, extant research suggests substantial social closure among black TGW due to existing residential segregation as well as the high levels of stigma and discrimination experienced by nonheteronormative individuals and individuals with HIV in predominantly black communities.^59,60^

In segregated or otherwise isolated neighborhoods such as those populated by many black TGW, social and sexual risks radiate and concentrate in an inward manner and become especially powerful, while simultaneously creating a need for community-building and expression. There is an extensive literature base asserting the instrumental resources, which ballroom/house communities and gay families, functioning as social networks, provide nonheteronormative black individuals.^28,61^ First popularized and emerging as a potent subculture during the Harlem renaissance of the 1920s, ballroom communities have facilitated rare opportunities for nonheteronormative black individuals to engage in sharing, comradery, and “being out.”^62^ Although research has shown that HIV and sexually transmitted infection (STI) risk may be more pronounced in the social networks characterizing these communities, these spaces simultaneously offer unique and promising opportunities for outreach and prevention efforts.^24,29,61^ In a recent analysis, researchers assessed the networks of ball-attending black MSM and TGW in the San Francisco Bay Area.^21^ Results from this investigation illustrated that participants with a high percentage of alters (i.e., the nodes to whom the ego is directly connected to) supportive of HIV testing were significantly more likely to have tested in the prior 6 months and less likely to have had condom-less anal sex in the prior 3 months.

In this analysis, we found provisional evidence that black TGW may conceptualize their sexuality differently over time, with these processes perhaps occurring, in part, as they feel more or less capable of expressing their broader social and gender identities. This finding tentatively suggests that there may be less a stable sexual milieu in this population than has been previously described, warranting further investigation. The fluidity in binary distinction in sexual identity may, at the same time, attenuate this group's gender identity.^63^ This may be due to the marked racial disparities in gender transition programs and the current trajectory of medicalization (e.g., puberty suppression),^64,65^ where black TGW transition later and are often gay-identified early on given limited options for other identification. In contrast to white TGW and TGW with higher income and levels of education, essential transition resources, such as puberty and hormone treatments and psychosocial support, may be substantially more difficult to access, financially and geographically, for black TGW. Moreover, recent research conducted among black TGW in Los Angeles^22^ has shown that this population is more likely to engage in hormone misuse as compared with nonblack TGW, with network analysis demonstrating that this risk may be more elevated among TGW with a greater number of hormone-using network alters.

This study supports the notion that maintaining social network salience and sustainability early on may be important in these identity-forming cultural spaces. As Graham observes,

Transgender identities may be perceived as more unstable, more in flux, and changing, whereas a gay identity may seem more certain; one is either gay or not, but the parallel perception may not exist for transgender identities.^15^

Results from our multiple-wave investigation further suggest that black TGW's experience is one punctuated by persistent socioeconomic and structural marginalization. In our assessment, only a slight majority of our participants, both TGW and MSM, had health care; furthermore, the majority of participants had little or no education behind high school, had an income of <$20,000 a year, and most of the TGW and over a quarter of the MSM indicated being unemployed. What is notable in our examination of network structure is that despite this socioeconomic isolation and pronounced sexual network differences, there were few differences in where TGW were positioned in the overall social network vis-à-vis their structural network position. This suggests that the social spaces of these young black men and women, and not individual-level behaviors, are very similar and worth examining in future work on health, resiliency, and well-being.

Another important observation from our study was the finding that TGW had more than threefold higher odds of receiving payment for sex. Formal and informal modes of sex work have been found to be especially pronounced among TGW, thereby greatly amplifying their risk for acquiring HIV and other sexually transmitted infections.^66–68^ However, a recent systematic review evidence confirms that there are currently very few evidence-based interventions focused on mitigating risk among the high proportion of sex workers in this population,^66^ highlighting the critical need for more concentrated attention in this area.^69^

There are some limitations to this study. First, the generalizability of our sample is limited due to the small portion of TGW who were ultimately recruited; moreover, the limited sample size may have contributed to issues related to statistical power. Furthermore, although the sample was representative of black individuals assigned male at birth who have sex with men, it is unclear how subgroups within this sample are represented, and thus we were unable to use RDS weights. Another limitation to this investigation is that we did not include a comparative sample of nonblack MSM, transgender men, or individuals from heteronormative populations, and thus it was not possible to discern to what extent our findings would have matched health and network patterns between nonheteronormative and heteronormative populations in other research contexts. Relatedly, another potentially valuable area of research that we did not explore in this analysis could be the social network dynamics between black TGW and black cis women. Finally, our sample included only younger individuals, and thus the findings here may not necessarily extend to older transgender or MSM groups, whose networks may be denser.^70,71^

In summary, the young nonheteronormative individuals in our sample were shown to face myriad barriers to health and well-being; these challenges were shown to manifest more densely among those in the study identifying as TGW. Network-focused interventions addressing the broad socioeconomic challenges in this population, and in particular the young black TGW subgroup, constitute a paramount component in efforts to improve the overall health outcomes and socioeconomic opportunities of those composing this vulnerable community. Particular attention should be placed on opportunities to infuse components into these interventions, which are both evidence based and culturally informed.

## Abbreviations Used

MSM: men who have sex with men
OR: odds ratio
RDS: respondent-driven sampling
SD: standard deviation
STI: sexually transmitted infection
TGW: transgender women
YBMSM: young black MSM
